# Examining the prevention approach in National Dementia Plans from European and North American countries

**DOI:** 10.3389/frdem.2024.1463837

**Published:** 2025-01-03

**Authors:** Mattia Andreoletti, Alessandro Blasimme

**Affiliations:** Department of Health Sciences and Technology, ETH Zurich, Zurich, Switzerland

**Keywords:** dementia, dementia prevention, National Dementia Strategy, Alzheimer's disease, prevention

## Abstract

**Objectives:**

This paper aims to provide a comprehensive review of National Dementia Plans (NDPs) from selected European and North American countries, focusing on the distinct prevention strategies outlined and the approaches employed for reducing dementia risk.

**Method:**

The sample consisted of 16 NDPs from Austria, Canada, Finland, France, Germany, Greece, Ireland, Italy, Liechtenstein, Luxembourg, Malta, the Netherlands, Spain, Switzerland, the UK, and the USA. These NDPs were retrieved from the Alzheimer's Disease International (ADI) database, with regular updates checked on official governmental websites. A qualitative analysis was conducted to identify common themes related to the vision, goals, and corresponding actions and measures within these strategies.

**Results:**

Our analysis revealed that dementia prevention is a strategic goal for most of the countries studied. Common actions identified include the identification of risk factors, advancing research, promoting healthy aging, increasing public awareness, and encouraging lifestyle interventions.

**Conclusion:**

We discuss the limitations and challenges of these actions, and more broadly, of the NDPs in relation to the recent literature on the most effective approaches to preventing dementia. We suggest adopting a more “horizontal” approach to dementia prevention, which current NDPs overlook in favor of “vertical” paradigms.

## 1 Introduction

As global life expectancy continues to rise and societies experience a demographic shift toward aging populations, the public health sector grapples with many challenges associated with age-related diseases, neurodegenerative disorders being particularly pressing. The World Health Organization (WHO) has indeed forecasted a considerable demographic shift, indicating that “by 2030, one in six people in the world will be aged 60 years or over. At this time the share of the population aged 60 years and over will increase from 1 billion in 2020 to 1.4 billion. By 2050, the world's population of people aged 60 years and older will double (2.1 billion). The number of persons aged 80 years or older is expected to triple between 2020 and 2050 to reach 426 million”.^1^ As societies continue to age, also the prevalence of dementia is anticipated to rise globally. The WHO estimates an escalation from 55 million individuals living with dementia in 2019 to a staggering 139 million by 2050 (World Health Organization, 2021). This surge not only presents a significant health challenge but also carries relevant economic implications. According to the World Alzheimer Report 2023 (Long et al., 2023), the financial burden of dementia is predicted to more than double, increasing from $1.3 trillion annually in 2019 to an astounding $2.8 trillion by 2030.

Until recently, efforts at developing a safe and effective treatment for dementia and for AD, have been frustrating and controversial despite conspicuous public and private investments. One notable example of controversy in developing a treatment for AD is the case of the drug aducanumab (Kesselheim, 2022). Nonetheless, the pursuit of finding a viable treatment continues to be a focal point for researchers and pharmaceutical companies worldwide, as the recent approval of Lecanemab demonstrates (Knopman and Hershey, 2023). Despite recent approvals of potentially disease modifying drugs, their effect sizes are small, and the trials are highly selective. So, prevention remains a priority (Burke et al., 2023; Leng and Yaffe, 2024; Walsh et al., 2022b).

For a long time, dementia was perceived as an unpreventable process of cognitive degradation and age-related decline, but in the last years emerging scientific findings have begun to challenge and reshape this paradigm. In 2017, the Lancet Dementia Prevention, Intervention, and Care Commission presented pivotal scientific evidence derived from observational, epidemiological, and interventional studies (Livingston et al., 2017). Such findings support an ambitious approach to dementia prevention, highlighting nine potentially modifiable risk factors across various aspects of lifestyle and health, which are believed to reduce the risk of developing dementia by over one-third. More recently, this list has been updated to include three additional key risk factors (Livingston et al., 2020). In 2024, two additional risk factors were added and 14 modifiable risk factors are now recognized which are: lower levels of education, hearing loss, hypertension, smoking, obesity, depression, physical inactivity, diabetes, excessive alcohol consumption, traumatic brain injury, air pollution, social isolation, high LDL cholesterol, and untreated vision loss (Livingston et al., 2024). A systematic review conducted by Deckers et al. (2015) identified a similar set of risk factors while adding hyperlipidemia to the list and suggesting considering coronary heart disease, renal dysfunction, diet and cognitive activity as potential additional risk factors, emphasizing the need for further investigation. In a recent cohort study (Dhana et al., 2024), a robust correlation has been revealed between maintaining a healthy lifestyle and sustaining cognitive performance until the end of life, irrespective of the burden of AD or related dementias. Also, recent studies, including a comprehensive meta-analysis by Yu et al. (2020), have identified several specific risk factors associated with AD, which accounts for ~70% of dementia cases. Notably, hyperhomocysteinemia has emerged as a significant risk factor with substantial evidence supporting its role in AD pathogenesis. Therefore, among potential preventive strategies, homocysteine-lowering treatments show particular promise.

Interestingly, some of the identified risk factors for dementia and AD are embedded in both social and environmental domains. In fact, addressing issues such as social isolation, facilitating early-life education, and combating air pollution necessitates political interventions at both local and global scales. Moreover, research on dementia prevention underscores also the significance of robust healthcare systems in reducing the prevalence and incidence of dementia over time. This highlights the intricate connection between dementia prevention and political, economic, and clinical considerations (Leibing and Schicktanz, 2021).

These findings and the dementia's global prevalence have prompted the WHO to designate it as a pressing public health concern, stressing the need for prevention strategies. The WHO's Global Action Plan on the Public Health Response to Dementia 2017–2025 emphasizes the importance of governments formulating specific national dementia policies by the year 2025, particularly in the action area of Dementia as a Public Health Priority (World Health Organization, 2017). In general, national public health strategies play a crucial role, especially in the realm of prevention (Wortmann, 2013). These strategies can encompass a spectrum of interventions, ranging from public health campaigns and awareness programs to targeted initiatives addressing risk factors associated with dementia. Furthermore, national strategies enable the allocation of resources and coordination of efforts across various sectors, such as healthcare, education, and social services. With regard to dementia, by tailoring approaches to the unique socio-cultural and healthcare landscapes of individual countries, national policies can effectively address the risk factors that determine the onset and the course of the disease. By focusing on prevention, national strategies have the potential to mitigate the rising burden of dementia on healthcare systems and improve the overall wellbeing of affected individuals and their families.

This paper aim is to provide a synthesis of National Dementia Plans (NDPs) from selected European and North American countries. The primary objective is to offer in-depth insights into the distinct prevention strategies articulated in these plans and the various approaches employed for the reduction of dementia risk. Then, we discuss our findings in relation to the recent literature on the most effective approaches to preventing dementia.

Through this exploration, we aim to provide a comprehensive understanding of the diverse measures and initiatives implemented by countries in addressing the challenges posed by the prevention of dementia.

## 2 Methodology

### 2.1 Data collection

Our data collection process focuses on acquiring relevant documents through the database maintained by the Alzheimer's Disease International (ADI)^2^ with regular checks for updates on official governmental websites to ensure the most current information is captured. Data collection was completed in February 2024.

### 2.2 Inclusion criteria

We included only NDPs from Western European countries and North America. This decision was motivated by two factors: limited resources preventing the inclusion of all global plans, and the desire to maintain a sample of comparable countries. Moreover, we had to limit our analysis to documents available in English, German, French, Spanish, and Italian, ensuring that at least one of the authors is proficient in the language for comprehensive understanding.

### 2.3 Data extraction and analysis

**Figure 1:**
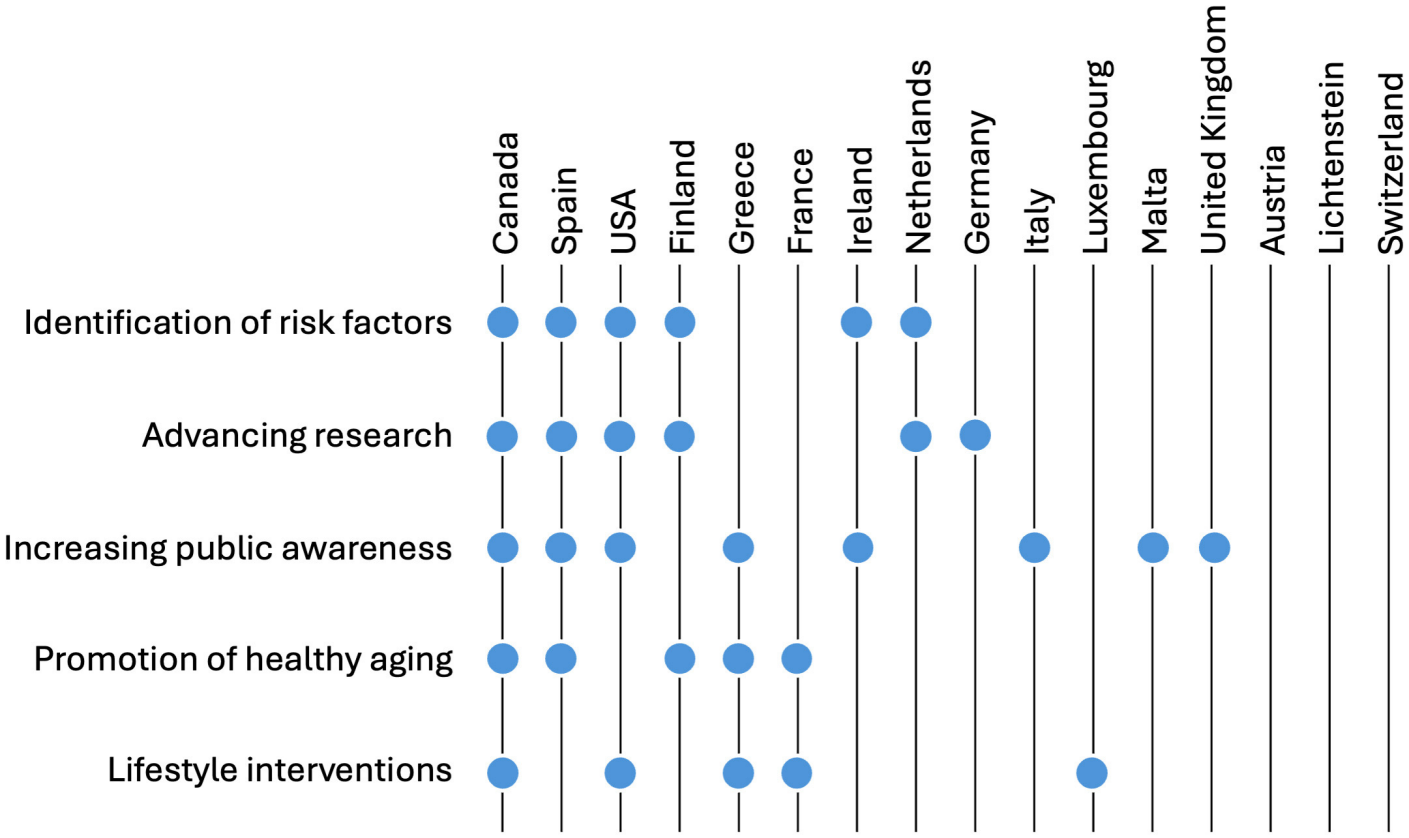
Themes and categories across national strategies.

For each selected country, we created extraction tables to identify the specific information to be gathered. This encompasses key details such as the title, year of publication, indication focus, and aims of the national strategy. Of particular interest is information on prevention, including specific programs if outlined, and the suggested approaches to dementia risk reduction. We reviewed all pertinent documents to extract insights on dementia prevention. The gathered information has been succinctly summarized in a comprehensive overview for each document (available in the Supplementary material). Additionally, a qualitative analysis (Braun and Clarke, 2006) was conducted to discern common themes related to the vision and goals of the strategies, along with the corresponding activities and measures. These findings were systematically clustered into broader categories (see Figure 1) and presented in a narrative format for a more straightforward understanding.

## 3 Results

### 3.1 Main characteristics

The ADI database had 39 documents available. For our analysis of dementia prevention within National Dementia Plans or strategies, we collected 16 documents from 16 countries, including Austria, Canada, Finland, France, Germany, Greece, Ireland, Italy, Liechtenstein, Luxembourg, Malta, Netherlands, Spain, Switzerland, the UK, and the USA.

These national plans have been published between 2009 and 2023, with the UK plan being the earliest and the US one standing as the most recently updated. These documents were developed and published by various governmental entities, with the ministries of health emerging as the predominant contributors (Table 1).

**Table 1:** Details of the included documents.

**Country**	**Title**	**Publisher**	**Year**
Austria	Dementia strategy. Living well with dementia	Federal Ministry of Social Affairs, Health, Care and Consumer Protection	2015
Canada	A dementia strategy for Canada. Together we aspire	Public Health Agency of Canada	2019
Finland	National Programme on Aging 2023. For an age-competent Finland	Ministry of Social Affairs and Health	2020
France	Neurodegenerative Diseases Roadmap 2021–2022	Ministry of Solidarity and Health	2021
Germany	National Dementia Strategy	Federal Ministry of Faimily Affairs, Senior Citizens, Woman and youth and Federal Ministry of Health	2020
Greece	National Action Plan for Dementia 2020	Ministry of Health	2017
Ireland	The Irish National Dementia Strategy	Department of Health	2014
Italy	National dementia plan—Strategies for promotion and improvement of the quality and appropriateness of healthcare interventions in dementia sector	Ministry of Health	2014
Liechtenstein	Dementia strategy for the Principality of Liechtenstein	Departments of Social Affairs and Health	2012
Luxembourg	Dementia Prevention Program-PDP	Ministry of Health	2015
Malta	Empowering change: A National Strategy for Dementia in the Maltese Islands 2015–2023	Parliamentary Secretariat for Rights of Persons with Disability and Active Aging	2015
Netherlands	National Dementia Strategy 2021–2030	Ministry of Health, Welfare and Sport	2020
Spain	Comprehensive Alzheimer's and other Dementia Plan (2019–2023)	Ministry of Health, Consumption and Social Welfare	2019
Switzerland	National Demetnia Strategy 2014–2019 (Results achieved in 2014–2016 and Priorities for 2017–2019)	Federal Department of Home Affairs FDHA and Federal Office of Public Health (FOPH)	2018
United Kingdom	Living well with dementia: A National Dementia Strategy. Accessible Summary	Department of Health	2009
United States	National Plan to Address Alzheimer's Disease: 2023 Update	Department of Health and Human Services	2023

Notably, the retrieved documents exhibit significant heterogeneity, showing many differences in terms of objectives, scopes, and level of detail on specific actions and implementation roadmaps. To demonstrate this variability, we can consider the two extremes: the Luxembourg's plan is a succinct 2-page focus on a specific dementia prevention program, whereas the US counterpart is an extensive 160-page document, which covers a wide range of topics.

### 3.2 Themes and categories

#### 3.2.1 Prevention as a strategic goal

Most of the countries recognize the prevention of dementia as a strategic goal of their national plans. Notably, some nations go further by explicitly designating it as a standalone objective.

For example, Canada emphasizes dementia prevention as the primary focus within its strategy. Similarly, Finland's strategy includes a comprehensive national program focused on aging, with a strong emphasis on preventing memory impairments. These countries expressly state that prevention is a critical component of their overall plans, recognizing the necessity for specific measures to minimize the prevalence of dementia.

In other cases, the emphasis on prevention is instead integrated into broader goals related to health promotion and heightened awareness. For example, Ireland places a high priority on raising knowledge of modifiable risk factors that influence the start and course of dementia, incorporating prevention into broader public health considerations. In Malta, although prevention is not explicitly stated as a goal, the national plan strategically focuses on improving awareness and understanding of dementia, with an emphasis on promoting help-seeking behaviors.

These various approaches demonstrate the global acknowledgment of the importance of dementia prevention, with countries implementing specialized policies based on their own healthcare systems and social interests. Whether expressly stated or integrated into broader health goals, the commitment to dementia prevention often reflects a widespread proactive approach to addressing the issues typically associated with cognitive health on both individual and social levels.

#### 3.2.2 Actions

Our analysis revealed several common actions in dementia prevention across different countries: identification of risk factors (six out of 16 countries), advancing research (6/16), increasing public awareness (8/16), promoting healthy aging (5/16), and lifestyle interventions (5/16). Canada's plan includes all these actions. Spain and the United States focus on four of them, Finland and Greece incorporate three. France, Ireland, and the Netherlands address two, and Germany, Italy, Luxembourg, Malta, and the UK cover one. Austria, Liechtenstein, and Switzerland prioritize instead the management and care of the disease over preventive measures (Figure 1). In the following sections, we present a synthesis of these various articulations related to dementia prevention found in the collected documents.

##### 3.2.2.1 Identification of risk factors

A recurring theme in the analyzed documents is the identification and evaluation of recognized risk factors. Interestingly, the United States, Finland, Ireland, Spain, the Netherlands, and Canada all demonstrate a similar dedication to preventative actions targeted at identifying and reducing dementia risk factors. The plans recognize that risk factors might differ among groups, which is why they frequently highlight the importance of tailored and individual approaches.

For example, groups may include older adults, individuals with a family history of dementia, or those with pre-existing health conditions such as hypertension or diabetes. Different approaches could involve targeted health education campaigns for older adults to promote cognitive health, community-based programs that address social isolation among at-risk populations, or personalized health assessments that consider genetic predispositions and lifestyle factors. The emphasis on identification goes beyond recognition and includes all-encompassing initiatives to incorporate research based on evidence into the creation of tailored preventive measures. The various countries show a shared commitment to developing efficient and focused interventions for lowering the total burden of dementia, at both the individual and societal levels, assigning a high priority on the identification of modifiable risk factors and putting preventive approaches based on scientific evidence into practice.

##### 3.2.2.2 Advancing research

In countries like Canada, Finland, Germany, Netherlands, Spain, and USA, dementia plans commit to advancing scientific research, supporting studies that focus not only on the identification of risk factors but also on the rigorous evaluation of targeted interventions designed to address these factors. The aim of research support is to provide the knowledge required to design interventions that not only demonstrate effectiveness but are also economically feasible in the pursuit of promoting cognitive health among aging populations. It is largely shared that building a solid body of evidence is a necessary first step in developing dementia prevention guidelines. These guidelines would then provide a framework that would enable the implementation of consistent and well-informed initiatives on a global scale.

##### 3.2.2.3 Increase public awareness

Another prevalent theme observed across the analyzed plans focuses on the significance of increasing public awareness about dementia, including the possibility of preventing it. Many strategies (Canada, Greece, Ireland, Malta, Spain, UK, and USA) strongly advocate for measures that empower individuals to enhance their understanding of dementia, its risk factors, and the importance of early intervention. These measures, which are part of a larger effort to prevent dementia, seek to foster a proactive mindset in society through education and participation initiatives. The focus on raising public awareness highlights how important educating communities is to addressing issues related to dementia prevention and cognitive wellbeing more in general.

##### 3.2.2.4 Promotion of healthy aging

A recurrent and overarching theme within national strategies is the promotion of active and healthy aging. Across many countries, such as Canada, France, Finland, Greece, and Spain, there is a clear commitment to cultivating an environment that fosters the wellbeing and vitality of aging populations. These strategies consistently emphasize the need of enhancing the functional capacity of older individuals, thereby extending the duration of active life years, and minimizing the need for intensive care and nursing. Innovative approaches are actively endorsed to motivate and support the older population in maintaining their health. These initiatives span a spectrum from nutritional improvements and increased physical activity to the promotion of mental health and social inclusion. The underlying goal is to create conditions that enable older individuals to age well, ensuring a high quality of life and overall cognitive health.

##### 3.2.2.5 Lifestyle interventions

Lifestyle interventions emerge as a major focus in several nations' National Dementia Plans, indicating a shared understanding of the critical role that individual behaviors and overall wellbeing play in lowering dementia risk. Canada, France, Greece, and the United States are notable examples of countries with preventive policies that emphasize a lifestyle approach. These countries highlight the importance of boosting physical activity, fostering health-focused behaviors, and encouraging overall wellbeing as key components of dementia prevention strategies. The emphasis on lifestyle interventions extends beyond general health advice, with a focus on the cognitive elements of aging. For example, Canada aligns its approach with the Lancet's framework, focusing on individualized risk factors, whereas France has a comprehensive plan that aims to incorporate preventive measures into many aspects of daily life, such as nutrition, physical activity, and smoking cessation. In this regard, the diversity of the national plans reflects the idea that dementia prevention requires more than just medical therapies, but also a coordinated effort to establish beneficial lifestyle modifications at both the individual and social levels. Through these initiatives, countries hope to enable individuals to make informed decisions that help to reduce dementia risk factors and improve general cognitive health.

## 4 Discussion

This study provides an exploration of national dementia strategies across diverse countries, shedding light on their perspectives and priorities regarding dementia prevention.

Previous reviews have consistently highlighted the lack of clarity in action plans within NDPs on a more general level (Seong et al., 2023; Wright and O'Connor, 2018). Our findings reinforce this observation, as we noted an insufficient formulation of specific action plans dedicated to targeting dementia prevention. The actions we identified remain mostly described in very general terms, lacking specific implementation plans. This raises concerns about the effectiveness of preventing dementia, especially given the multi-domain nature of interventions often required. While the potential effectiveness of policies and interventions has been demonstrated in research settings, further research, such as high-quality cost-effectiveness evaluations (Braun et al., 2024; Handels and Wimo, 2019; McRae et al., 2020), and well-designed multicenter Randomized Controlled Trials (RCTs; e.g., Rosenberg et al., 2018) are likely needed to provide robust support and influence policymakers to take more decisive actions. This is evident in the commitment of many countries to support further scientific research, focusing not only on identifying risk factors but also on rigorously evaluating targeted interventions designed to address them.

Interestingly, raising public awareness about the possibility of preventing dementia is the most frequently mentioned action in the documents. This is consistent with the WHO Global Action Plan on Dementia: “Risk reduction of cognitive decline and dementia” that urged all countries to have “at least one functioning public awareness campaign on dementia to foster a dementia-inclusive society by 2025” (World Health Organization, 2017, p. 15). And many countries recognized the importance of informing the public also about the possibility of prevention. This is a necessary first step to effectively address the prevention of the disease. Indeed, a systematic review published in 2018 indicated that public understanding of the potential for dementia prevention is still limited. Most importantly, the significance of cardiovascular health in dementia prevention was not widely understood (Cations et al., 2018). And raising awareness about this topic is critical to promote lifestyle interventions that address the related risk factors (Steyaert et al., 2021).

Our research also highlights a prevailing emphasis in national plans on prioritizing lifestyle interventions, once again this is consistent with the WHO guidelines (World Health Organization, 2019) and the Global Action Plan on dementia (World Health Organization, 2017), both advocating for individual risk-reduction interventions targeting behavioral changes. However, in the scientific literature, there are many concerns about the limitations of relying solely on individual interventions, particularly in addressing the long-term burden of diseases like dementia (Walsh et al., 2022a). In response to these challenges, there is a call for a strategic redirection of interventions to address the burden of dementia more effectively. This strategic shift involves reorienting the focus toward societal determinants, such as poverty, income inequality, and publicly funded health services. This perspective suggests expanding intervention strategies beyond individual behavioral changes, urging for population-wide approaches that acknowledge the multi-domain nature of dementia prevention (Walsh et al., 2023b, 2024). Population-level interventions aim to modify the distribution of risk factors across the entire populace by addressing societal and structural conditions influencing the development and persistence of these factors. These broader approaches often result in more substantial reductions in disease incidence and prevalence, foster greater health equity, and produce longer-lasting effects compared to individual-level strategies. By broadening the scope of preventive policies, there is an opportunity to enhance the equity and efficiency of interventions, aligning with the documented benefits of the population-level approach in existing literature. In this regard, Walsh et al. (2024) systematically identified 26 population-level interventions that are supported by moderate to high scientific evidence, specifically targeting the reduction of established risk factors for dementia. These interventions can be implemented through various strategies, including fiscal, marketing, availability, and legislative measures.

However, this kind of population-level approach is not found in the retrieved NDPs. The reason may lie in scientific and political challenges in terms of research and implementation, often leading to inadequate exploration (Walsh et al., 2023a). Scientific challenges include for instance the need for interdisciplinary collaboration and funding for large-scale studies that encompass diverse populations and risk factors. Politically, there may be resistance to implementing population-level interventions due to competing public priorities and short-term political cycles.

According to our understanding and interpretation of the NDPs, preventive strategies are often designed in fragmented and disjointed manners, with national approaches primarily focusing on preventing individual diseases through what can be labeled as “vertical” preventive paradigms. Vertical preventive paradigms are public health programs specifically targeting the prevention of a single disease, such as a national campaign aimed solely at reducing the incidence of diabetes. These programs typically focus on interventions that directly address risk factors and behaviors associated with the targeted disease, without necessarily considering broader health outcomes or addressing common risk factors across multiple conditions. However, recent insights have emphasized that many risk factors for dementia are also significant for a wide range of other conditions, such as cardiovascular diseases (Brain et al., 2023). This recognition calls for a shift toward more “horizontal” approaches, like the life-course approach (Chapko et al., 2018; Mikkelsen et al., 2019) or ground-state prevention (Blasimme, 2021; Blasimme et al., 2021). These approaches to prevention take instead a broader view and address common risk factors across multiple diseases. As such, they are characterized by being disease-agnostic and catering to the needs of diverse groups (e.g., age groups). On this account, it is fundamental to establish connections between dementia prevention efforts and existing primary prevention strategies, such as initiatives promoting active and healthy aging or brain health (Hussenoeder and Riedel-Heller, 2018). Doing so would enable a more holistic and longitudinal approach to prevention, aligning with recent efforts by the recent WHO report on optimizing brain health (World Health Organization, 2022).

### 4.1 Limitations

The exclusion of sub-national plans may underestimate the comprehensive landscape of dementia-related initiatives, especially in countries with substantial local autonomy, such as Canada or Switzerland (Andreoletti and Blasimme, 2024). We also caution that the mere presence of a policy plan does not guarantee successful implementation, emphasizing the need for specificity, cooperation among policy actors, effective evaluation mechanisms, and appropriate budget allocations for policy success. Moreover, some countries whose NDPs do not include population-level actions on risk factors may have comprehensive plans within their Non-Communicable Disease (NCD) strategies. However, these plans are not labeled as dementia prevention and are therefore not included in the dementia plan. Further research is needed to investigate this.

It is also important to note that our study did not include Low- and Middle-Income Countries (LMICs). This exclusion may restrict the applicability of our findings to regions with different economic contexts and healthcare infrastructures. Consequently, the insights gained from our study may not fully capture the diverse range of challenges and approaches to dementia prevention strategies in LMICs, which often face unique resource constraints and public health priorities. Further research focusing on LMICs is therefore warranted to better understand the nuances of dementia prevention strategies in diverse socioeconomic contexts (Jeraldo et al., 2023).

Despite these limitations, our study provides valuable insights for policymakers and researchers, serving as a practical reference for the development, updating, and evaluation of dementia prevention strategies within NDPs.

## Data Availability

The original contributions presented in the study are included in the article/Supplementary material, further inquiries can be directed to the corresponding author.

## Footnotes

^1^https://www.who.int/news-room/fact-sheets/detail/ageing-and-health. Accessed 7/11/2024.

^2^https://www.alzint.org/what-we-do/policy/dementia-plans/. Last access on 27/02/2024.
