# Supplementary material for: Examining the prevention approach in National Dementia Plans from European and North American countries
Source: Front Dement. 2025 Jan 3;3:1463837. doi: 10.3389/frdem.2024.1463837 (PMC11738615; doi:10.3389/frdem.2024.1463837)
Supplement: Supplementary file 1 [file Table_1.DOCX]

**Overview of country-specific-strategies**

**Austria**

Austrian strategy on dementia explicitly leaves aside the theme of dementia prevention. Nevertheless, the strategy recognizes its significance and commits to incorporating research findings into its implementation promptly as they become available.

**Canada**

In Canada, the National Dementia Strategy (NDS) places a significant emphasis on dementia prevention as the primary focus among its three national objectives. The overarching goal of the strategy is to address the growing burden associated with cognitive health in aging adults by prioritizing the prevention of dementia, advancing therapies and seeking a cure, and improving overall quality of life.

Specifically, Canada's strategy aims to enhance dementia prevention by identifying and assessing risk factors. This involves a concerted effort to increase awareness about behavioral changes, protective factors, and effective interventions. Aligned with the Lancet reports from 2017 and 2020, the Canadian approach to dementia prevention is rooted in addressing individualized risk factors, with a strong focus on identifying potential risks and reinstating healthy behaviors. The Canadian NDS acknowledges the necessity for further research and evidence-based interventions to deepen the understanding of dementia risk factors. It underscores the importance of exploring how individual and collective choices can play a role in reducing the risk of dementia. The strategy also highlights the need for increased education among healthcare professionals and the public regarding identifiable risk factors and the positive impact of healthy lifestyles in mitigating the risk of dementia.

**Finland**

Finland's forward-looking strategy on dementia prevention, encapsulated in its “Objectives 2030”, reflects a dedicated national program focused on aging. With a resolute commitment to enhancing the well-being of older individuals, Finland places a significant emphasis on preventing memory disorders. A cornerstone of this strategy is the widespread adoption of the FINGER model [10], renowned for its comprehensive framework. This model systematically addresses the prevention of chronic diseases prevalent in Finland, with a special emphasis on preserving functional capacity, particularly cognitive function. The proactive approach adopted by the country not only serves to reduce the incidence of memory disorders but also plays a pivotal role in minimizing the need for related services.

**France**

France has integrated the prevention of neurodegenerative diseases, including Alzheimer’s disease and dementia, as a pivotal element within its broader strategy. This commitment encompasses a comprehensive set of preventive actions grounded in life course determinants. Emphasizing holistic well-being, initiatives range from promoting physical activity and fostering health-centric schools to addressing hearing risks among the youth, implementing treatments to aid smoking cessation, and executing a national program to combat tobacco use. Noteworthy efforts include the extension of Nutriscore, reduction of salt consumption, and the overarching Public Health Nutrition and Health Program (PNNS).

Within this framework, paramount attention is given to the triad of food, nutrition, and physical activity, underscored by actions initiated in 2019. The focus on “Aging well and preventing loss of autonomy” involves collective prevention measures, fortified by the establishment of conferences dedicated to preventing loss of autonomy. This approach includes a national undernutrition week, targeted awareness campaigns for seniors regarding the benefits of physical activity and healthy eating (featuring workshops and sports courses), and encouragement for physicians to assess physical condition.

Innovative tools augment these efforts, such as the “Advancing in Good Health” health application. This platform, scheduled for implementation by the end of 2021, facilitates self-assessment, offers operational advice, and provides personalized guidance for reinforced prevention in the age group of 40-45, as orchestrated by Public Health France.

The strategy extends its reach to individuals in the transition to retirement through “meetings for young retirees,” aiming to engage 200,000 people annually in prevention meetings until 2022. Furthermore, a multidimensional screening program, aligned with the WHO’s Integrate Care for Older People approach (ICOPE), encompasses locomotion, nutrition, hearing, vision, mental health, and cognition.

Other targeted measures include the dissemination of messages guiding the identification and management of hearing loss, facilitated by the provision of a self-test by the Hearing Foundation. Additionally, learned societies of ophthalmologists are spearheading the definition of benchmarks for screening vision disorders associated with aging, with plans for implementation dating back to 2021.

**Germany**

The German government aims to impkement a comprehensive approach to dementia prevention, focusing on lifestyle factors through initiatives like IN FORM and the "Alcohol? Know Your Limit" campaign. GKV-SV guidelines and the Prevention Act provide a foundation, emphasizing the nutrition-dementia link. Health literacy is prioritized for public education. For example, the GKV-Bündnis für Gesundheit initiative at the municipal level allocates substantial funding to promote health and disease prevention, particularly benefiting vulnerable groups, including those with dementia.

Recognizing the lack of effective treatments, the German strategy advocates extensive research through collaborations between research, industry, and regulatory authorities, with a focus on engaging scientific start-ups. The strategy explictly supports clinical research for biomarker development and predictive diagnostics. Initiatives like MonAge and surveys like DEAS contribute valuable information. Aslo, the strategy aims to establish a National Research Data Infrastructure for systematic access to digital databases, fostering collaboration for meaningful research data management.

The document acknowledges the importance of developing strategies for secondary and tertiary prevention, focusing on real-life scenarios, maintaining autonomy, and supporting family caregivers. These strategies aim to prevent complications and concomitant diseases (secondary prevention) and rehabilitate functional skills (tertiary prevention). Digitalization is highlighted for improving dementia prevention and health services. Notably, ethical considerations in dementia research are underscored, promoting stakeholder involvement, collaboration, and a broad approach in supporting evidence-based strategies and prevention measures in dementia care.

**Greece**

In Greece, the strategic focus on preventing dementia is deeply intertwined with initiatives aimed at informing the population and fostering public awareness. Recognized as one of the seven essential axes within the national dementia strategy, the Greek approach unfolds through two key actions. The first action involves an intervention for dementia prevention, concentrating on widespread education and the implementation of preventive programs. With objectives centered on informing the public and diminishing the occurrence of dementia, collaborative efforts between Memory and Mental Functions Clinics, Non-Profit Organizations for Alzheimer's, and various stakeholders are paramount. The second action revolves around disseminating information and cultivating public awareness to facilitate early diagnosis and treatment, while simultaneously combating the associated stigma. This comprehensive strategy includes nationwide information campaigns, local events, publication of informative brochures, and targeted training for professionals in contact with individuals affected by dementia.

**Ireland**

The Irish strategy places a strong emphasis on cultivating awareness of modifiable risk factors influencing the onset and progression of dementia. Acknowledging the potential to mitigate the risk of developing dementia, the strategy draws attention to recent epidemiological findings about modifiable risk factors. It underscores the impact of lifestyle choices, such as smoking, obesity, and physical inactivity, on cardiovascular health, which, in turn, can influence dementia risk. Moreover, it acknowledges the importance of non-modifiable risk factors, such as age, gender, and genetics. Finally, the strategy emphasizes the need for primary prevention strategies and heightened public awareness, highlighting the crucial role of proactive measures in addressing the complexities of dementia.

**Italy**

The Italian dementia strategy adopts a comprehensive approach, with a dual focus on scientific research and the establishment of an integrated network for the management of dementia, encompassing prevention. Recognizing the pressing need to advance scientific understanding, particularly in the realm of prevention, the strategy places special emphasis on evidence directly applicable to public health. The overarching goal is to facilitate prevention, early diagnosis, and subsequently, appropriate treatment. The plan targets increased awareness among various stakeholder groups, including the general population, individuals with dementia, their families, and healthcare professionals. Additionally, it prioritizes the establishment of an integrated network to streamline prevention and management efforts, fostering collaboration between different institutions and organizations.

**Luxembourg**

The Luxembourg Dementia Prevention Program (PDP) is a comprehensive initiative designed to enhance the quality of life for individuals in the early stages of Alzheimer's disease or vascular dementia. Recognizing that the onset of dementia can be diagnosed well before its symptoms manifest, the program focuses on timely intervention to delay or prevent the progression of the disease. PDP offers personalized preventive measures, combining nutrition, physical and social activities, cognitive training, and medical and non-medical interventions. Tailored to each participant's individual requirements and risk factors, the program aims to empower individuals to proactively manage their health. The initiative provides ongoing support, addressing not only medical aspects but also assisting in life planning and answering detailed questions about the diagnosis. Eligible participants include those with a confirmed early diagnosis of dementia, and participation is voluntary upon the treating physician's request. The program collaborates with local providers and is free for patients. While preventive measures may not guarantee success for everyone, PDP equips individuals with the knowledge and resources to navigate the challenges associated with dementia. It also emphasizes the importance of early diagnosis, providing information on medications and supporting the transition to care in collaboration with relevant institutions.

**Malta**

Malta's national dementia strategy does not explicitly recognize prevention as a stated goal. Nonetheless, it underscores the importance of elevating awareness and understanding of dementia among both the general public and healthcare professionals. This strategic approach aims to diminish stigma and dispel misconceptions surrounding the condition. Central to this effort are information campaigns designed to disseminate knowledge about dementia, preventive measures, the significance of timely diagnosis, and the array of available community and support services. The strategy extends its educational reach to non-professional sectors, ensuring that service providers in direct contact with individuals affected by dementia are well-informed. The primary goal is to foster increased awareness and understanding among the public and healthcare professionals, emphasizing the encouragement of help-seeking behavior. The strategy outlines continuing information campaigns targeting diverse demographics, using various media channels. In principle these educational campaigns will provide comprehensive information on the risk factors and preventive measures.

**Netherlands**

The Netherlands' dementia strategy addresses prevention within its dedicated research part. This strategy allocates resources to support fundamental research across various themes, specifically emphasizing prevention. Acquiring insights into possibilities for reducing the risk of dementia encompasses both basic research and investigations into the effects of lifestyle factors. Notably, a distinct "risk reduction" theme is set to be integrated into the upcoming dementia research program initiated by The Netherlands Organization for Health Research and Development (ZONMW). In collaboration with the Dutch Research Council (NOW), the document provides for an evaluation to explore the incorporation of this theme into the research activities of these organizations. This collaborative effort aims to foster a well-coordinated and expansive research initiative centered on diminishing the risk of dementia.

**Spain**

Spain's national strategy for dementia includes AD and dementia prevention, alongside treatment and diagnosis. Spain's objectives are firmly rooted in integrating AD and dementia prevention into existing health campaigns, especially those aimed at reducing the risk of chronic diseases and fostering general health promotion and active aging. This integration is carried out through the deployment of the Health Promotion and Prevention Strategy of the National Health System under the Ministry of Health, Consumption, and Social Welfare.

A pivotal aspect of Spain's primary prevention focus is the promotion of the identification of individuals at high risk of Alzheimer's. To achieve this, the country is undertaking a comprehensive review, with the aim of disseminating knowledge about risk factors within the primary care domain. This review not only takes stock of the current state of science on Alzheimer's but also outlines measures that can potentially mitigate such risks, aligning with the promotion of healthy lifestyles and evidence-based interventions.

Within the multifaceted lines of action, Spain is actively incorporating the reduction of Alzheimer's risk as a specific objective in various programs and policies. A comprehensive review of dementia prevention interventions forms a cornerstone of this effort, with the subsequent publication of a guide based on the findings. This guide not only serves as a knowledge repository but also offers actionable recommendations rooted in scientific evidence.

Spain's commitment extends further to the design, promotion, and financial support of pilot studies evaluating the feasibility and effectiveness of preventive interventions against Alzheimer's. These studies are deemed essential in shaping evidence-based practices and refining strategies to advance dementia prevention initiatives. In parallel, tools such as the dementia risk index have been disseminated among primary care professionals and citizens, in order to a proactive engagement in risk identification and mitigation.

**UK**

The UK strategy addresses dementia prevention only within the objective of raising awareness and promoting early intervention. The envisioned outcome is a heightened awareness and understanding of dementia among both the public and professionals. This increased awareness is intended to facilitate a better comprehension of the advantages associated with early diagnosis and care, ultimately encouraging proactive measures for the prevention of dementia. No further specifications are given.

**USA**

The United States has a multifaceted national plan to address dementia and Alzheimer’s disease. Prevention, together with treatment are recognized as the main goals. This goal underscores the commitment to expanding research efforts aimed at understanding the causes, prevention, and treatment of AD and related dementias. The objective is to develop additional prevention and treatment modalities by 2025, aligning with ongoing research and clinical inquiry. The strategy emphasizes prioritizing and accelerating scientific research to swiftly translate evidence-based solutions into practical interventions. The Department of Health and Human Services (HHS) aims to set interim milestones and ambitious deadlines to ensure the timely implementation and scaling of effective strategies, enabling individuals with AD or dementia to benefit from advancements in scientific knowledge.

With regard to prevention, one goal focuses specifically on accelerating public health action to address risk factors for AD and related dementias. The strategy recognizes the significance of public health in protecting and promoting the well-being of communities. The Centers for Disease Control and Prevention (CDC) play a crucial role in convening summits, disseminating information, and educating public health workforces on risk factors. A key initiative involves collaborating with various organizations to create a network for tailored messaging and interventions, targeting different populations, such as Black, Hispanic, and Tribal communities.

Another action focuses on accelerating the dissemination of information on risk reduction to public health entities. This includes developing resources and conducting webinars to promote the importance of public health in addressing brain health.

Education is a central component of the strategy, with the CDC developing a comprehensive Public Health Curriculum to address cognitive health, cognitive impairment, and dementia. Furthermore, the strategy emphasizes the importance of physical activity in preventing AD and related dementias, aligning with the Physical Activity Guidelines for Americans. Initiatives like expanding access to evidence-based health promotion and disease prevention programs through the Aging Network aim to engage older adults in adopting healthier lifestyles.

Recognizing disparities in risk factors among marginalized populations, the strategy includes actions tailored to address these inequities. This involves supporting the development of culturally sensitive programs and materials to increase awareness of brain health, particularly for African American and Tribal communities. The National Brain Health Center for African Americans (NBHCAA) is actively involved in raising awareness through partnerships with faith-based institutions and health professionals.
